# Incidence, causes and consequences of moderate and severe traumatic brain injury as determined by Abbreviated Injury Score in the Netherlands

**DOI:** 10.1038/s41598-021-99484-6

**Published:** 2021-10-07

**Authors:** Denise Jochems, Eveline van Rein, Menco Niemeijer, Mark van Heijl, Michael A. van Es, Tanja Nijboer, Luke P. H. Leenen, R. Marijn Houwert, Karlijn J. P. van Wessem

**Affiliations:** 1 Department of Surgery, University Medical Center Utrecht, PO Box 85500, 3508 GA Utrecht, The Netherlands; 2 Department of Neurology, University Medical Center Utrecht and de Hoogstraat Rehabilitation, Utrecht, The Netherlands; 3 Department of Experimental Psychology, Helmholtz Institute, Utrecht University, Utrecht, The Netherlands; 4 Center of Excellence for Rehabilitation Medicine, UMC Utrecht Brain Center, University Medical Center Utrecht, and De Hoogstraat Rehabilitation, Utrecht, The Netherlands

**Keywords:** Neurology, Trauma

## Abstract

Traumatic brain injury (TBI) is a leading cause of death and disability. Epidemiology seems to be changing. TBIs are increasingly caused by falls amongst elderly, whilst we see less polytrauma due to road traffic accidents (RTA). Data on epidemiology is essential to target prevention strategies. A nationwide retrospective cohort study was conducted. The Dutch National Trauma Database was used to identify all patients over 17 years old who were admitted to a hospital with moderate and severe TBI (AIS ≥ 3) in the Netherlands from January 2015 until December 2017. Subgroup analyses were done for the elderly and polytrauma patients. 12,295 patients were included in this study. The incidence of moderate and severe TBI was 30/100.000 person-years, 13% of whom died. Median age was 65 years and falls were the most common trauma mechanism, followed by RTAs. Amongst elderly, RTAs consisted mostly of bicycle accidents. Mortality rates were higher for elderly (18%) and polytrauma patients (24%). In this national database more elderly patients who most often sustained the injury due to a fall or an RTA were seen. Bicycle accidents were very frequent, suggesting prevention could be an important aspect in order to decrease morbidity and mortality.

## Introduction

Traumatic brain injury (TBI) is a growing global health problem; it is a leading cause of death and life-long disability^1,2^. In 2012, approximately 57,000 deaths (11.2/100,000) in the European Union were estimated to be related to TBI, in 2010 almost 53,000 deaths (17.7/100,000) were attributed to TBI in the United States^2,3^. In the Netherlands, there was a significant rise in admissions for and presentations with TBI to the accident and emergency department (A&E) between 1998 and 2012^4^. Furthermore, TBI is the main cause of death in severely injured trauma patients and contributes to at least 30% of deaths caused by trauma^5–8^.

The overall incidence of TBI is increasing with changing epidemiology, where causes of injury seem to depend on the status of development of the country. In low- and middle-income countries road traffic accidents (RTA) are the main cause of TBI, as motorised traffic is more common and safety rules are lacking. However, in high-income countries, the number of elderly patients with a brain injury due to a fall is rising, whereas preventative measures have decreased the number of TBIs due to road traffic accidents^3,6,7,9,10^. Unfortunately, the precise global incidence is unknown due to a lack of data collection and comprehensive studies on the subject^8,11^*.*

Data on epidemiology is important for healthcare policies on where to target prevention strategies. Recent literature on changing epidemiology of TBI in Western Europe^4^ shows that despite the increase in incidence, mortality rates remain stable. A possible explanation could be that low-energy falls are less likely to cause death than RTAs, which are more likely to cause polytrauma. Furthermore, TBI in elderly is more likely to be caused by a fall^2,4^. All grades of TBI were included in these studies. Few studies focus on moderate and severe TBI. Different definitions of severity of TBI, such as Glasgow Coma Scale scores at presentation and Intensive Care Unit (ICU) admission, make comparison difficult. Furthermore, pre-hospital intubation and intoxication can complicate these scores, which can lead to inclusion of mild TBI in analysis^12,13^. The Abbreviated Injury Score (AIS) is an established standardised score for injuries based on probability of survival with this injury, which allows for accurate classification and comparison of TBI^14^.

Moderate and severe TBI are more likely to lead to mortality and poor functional outcomes than mild TBI. Therefore, data on epidemiology of true moderate and severe TBI could provide more insight into where to focus research and prevention methods in order to decrease poor outcome. Especially, when focussed on important groups: polytrauma patients and the elderly, as the hypothesis is that the first seems to contribute mainly to the increasing incidence and the latter to mortality.

The aim of this nationwide study was to describe the incidence, distribution of age, causes, and consequences of moderate and severe TBI for the whole population and in particular polytrauma patients and elderly.

## Methods

For this nationwide retrospective cohort study of patients who were admitted with moderate or severe TBI, data was collected from the Dutch Trauma Registry (DTR). The registry is an excellent representation of Dutch trauma care, as 99% of hospitals contribute to the DTR. The aim of the DTR is to uphold and monitor good standard of care for injured patients. The DTR has been used for nationwide retrospective cohort studies before, such as Peek et al.^15^. The DTR contains data of all trauma patients who were admitted to hospital through A&E, within 48 h of trauma. Patients who die prior to arrival in A&E or do not have to be admitted, are not kept in the database. This also applies to patients who are admitted, but not due to their traumatic injuries^15,16^. National demographic data were obtained, from the Dutch Population Register from the *Central Bureau of Statistics* to determine incidence rate of moderate and severe TBI requiring hospital admission^17^.

All patients aged 17 years and older admitted to the hospital between January 2015 and December 2017 with moderate or severe TBI were identified using Abbreviated Injury Scale (AIS) codes for traumatic brain injury. The AIS is a widely accepted anatomically based scoring system to grade injuries from mild to maximal (almost certainly leading to death) on a scale from one to six and raters can use data from the patient’s records to assign a score, using a supplied standardised guideline^18^. Combined AIS scores are used to determine the Injury Severity Score (ISS). AIS scores as recorded in the DTR were calculated by data managers of the participating trauma centres as per ISS 08^19^.

Moderate to severe TBI was classified as an AIS of the head region (AIShead) of three or higher. Two subgroups of polytrauma and elderly were analysed separately as well. Polytrauma was defined as an Injury Severity Score (ISS) of 16 or higher. Elderly patients were defined as patients aged 65 years and older. To prevent inclusion of duplicate cases, all patients who required early transfers to another hospital were excluded.

The following baseline variables were obtained from the DTR: age at trauma, sex, American Society of Anesthesiologists (ASA) score, mechanism of injury, Glasgow Coma Scale (GCS), AIS scores for all body regions, Injury Severity Scores (ISS) scores and Revised Trauma Score (RTS). GCS was evaluated in the A&E department in all cases and only noted if all three parameters (eye, motor and voice) were available. In addition, if the patient was intubated and sedated prior to GCS scoring, their GCS score was not included in analyses. The DTR does not include data on who recorded the GCS score. The Revised Trauma Score (RTS) is a widely used scoring tool to determine the initial trauma severity based on the GCS, systolic blood pressure, and respiratory rate. A lower score reflects a higher severity of injury^20^.

The in-hospital treatment variables obtained were: activation of trauma team in hospital, involvement of the Mobile Medical Team (MMT), need for emergency intervention and highest level of received care. In the Netherlands, the MMT consists of a trauma surgeon or anaesthetist and a trained nurse to provide acute care at the site of the accident.

The in-hospital outcome variables obtained were hospital length of stay (H-LOS), ICU length of stay (ICU-LOS), mortality, H-LOS until death and Glasgow Outcome Scale (GOS) score at the time of hospital discharge.

All variables were collected for all included patients and separately for polytrauma patients and the elderly.

Frequencies with percentages were used to describe categorical data.. The Shapiro–Wilk test and Quantile–Quantile plots confirmed whether data were normally distributed or not. Descriptive data included means with standard deviations (SD) for normally distributed continuous data and medians with interquartile ranges (IQR) for non-normally distributed continuous data. The incidence rate was calculated by dividing the total number of patients with TBI by the total Dutch population ≥ 16 years of age or > 64 for the elderly for the inclusion period. Incidence rates were presented per 100,000 person-years. For statistical analyses, SPSS statistical software (SPSS 23.0; IBM Inc., Armonk, NY, USA) was used.

The Medical Ethical Review Board of the University Medical Center Utrecht approved this study and granted a waiver of informed consent (WAG/mb/18/011,787). All methods were performed in accordance with the relevant guidelines and regulations.

## Results

In total, 12,650 adult patients with moderate or severe TBI were admitted to Dutch hospitals between January 2015 and December 2017 . Of all patients, 355 were excluded from analysis due to early transfer, leaving 12,295 for analysis.

On 1 January 2016, 16,979,120 people lived in the Netherlands, of whom 13,766,208 (81%) were 17 or older and 3,107,842 (18%) were 65 or older.

The incidence rate of moderate or severe TBI was 30 per 100,000 person-years. Patients in our cohort had a median age of 65 years (IQR: 47–79). Patients were predominantly male (n = 7,482; 61%). Median American Society of Anesthesiologists (ASA) score was 2 (IQR: 1–2), median AIShead was 3 (IQR: 3–4) (Table 1).

**Table 1:** Baseline variables.

	All	Polytrauma	Elderly
	n = 12,295	n = 5,763 (47%)	n = 6,228 (51%)
**Demographics**			
Incidence rate *person years*	30/100,000	14/100,000	67/100,000
	**Median (IQR)**	**Median (IQR)**	**Median (IQR)**
Male sex	7,482 (61)	2,671 (64)	3,264 (52)
	**N (%)**	**N (%)**	**N (%)**
Median age	65 (47–79)	64 (45–78)	79 (72–85)
Clinical characteristics	**Median (IQR)**	**Median (IQR)**	**Median (IQR)**
Median ASA	2 (1–2)	2 (1–2)	2 (2–3)
*Missing*	*2,093 (17)*	*963 (17)*	*997 (16)*
Median AIShead	3 (3–4)	4 (3–5)	3 (3–4)
RTS A&E	7.8 (6.9–7.8)	7.8 (5.0–7.8)	7.8 (7.6–7.8)
*Missing*	*4,722 (38)*	*2,137 (37)*	*2,449 (39)*
	**N (%)**	**N (%)**	**N (%)**
ISS > 16	5,763 (47)	5,763 (100)	2,775 (45)
**AIShead**			
3	7,778 (63)	1,731 (30)	3,856 (62)
4	2,486 (20)	2,001 (35)	1,262 (20)
5 + 6	1,997 (16)	2,031 (35)	1,110 (18)
**Trauma mechanism**			
Fall	5,579 (52)	2,533 (44)	3,543 (57)
Low *(% of falls)*	4,245 (76)	1,662 (63)	3,013 (85)
RTA	4,328 (40)	2,249 (39)	1,574 (25)
Bicycle *(% of RTAs)*	2,523 (59)	1,152 (51)	1,155 (73)
*Missing*	*1547 (13)*	*603 (10)*	*866 (14)*
GCS 15	5,483 (60)	1,746 (45)	3,023 (63)
*Missing*	*3,067 (25)*	*1,926 (33)*	*1,360 (22)*

IQR = Interquartile range, ASA = American Society of Anaesthesiologists score, AIShead = Abbreviated Injury Scale of the head region, ISS = Injury Severity Score, RTA = Road traffic accident, GCS = Glasgow Coma Scale score, RTS = Revised Trauma Score.

Falls were the most common trauma mechanism (n = 5,579; 52%), closely followed by road traffic accidents (n = 4,328; 40%). Falls from low height accounted for 76% of all falls. RTAs included cyclists, who accounted for the, by far, largest proportion of this group (n = 2,523; 59% of all RTAs), followed by accidents with mopeds (n = 632, 15%) and motorised vehicles with more than two wheels (n = 627, 14%). Less frequent were accidents where the victim was a pedestrian (n = 355, 8%) or motorcyclist (n = 108, 2%). GCS scores were missing for a quarter of patients. GCS of 15 was noted in 5,483 (60%) patients. Median RTS in A&E was 7.8 (IQR: 6.9–7.8) (Table 1).

MMT and in-hospital trauma team were involved in, 1,856 (15%) and 4,255 (42%) of cases, respectively. Of all patients, 1,045 (10%) underwent an emergency intervention, mostly neurosurgical procedures (n = 716; 69%). Highest level of care was most often the ward (n = 6,323; 56%) (Table 2).

**Table 2:** Treatment variables.

	All	Polytrauma	Elderly
	n = 12,295	n = 5,763	n = 6,228
	**N (%)**	**N (%)**	**N (%)**
Involvement MMT	1,856 (15)	1,514 (26)	568 (10)
*Missing*	*526 (4)*	*155 (3)*	*304 (5)*
Trauma team activated	4,255 (42)	3,161 (62)	1,549 (31)
*Missing*	*2,045 (17)*	*653 (11)*	*1,194 (19)*
Emergency intervention	1,045 (10)	989 (18)	272 (5)
Craniotomy *(% of intervention)*	448 (43)	418 (42)	142 (52)
ICP *(% of intervention)*	268 (26)	248 (25)	48 (18)
*Missing*	*1,336 (11)*	*404 (7)*	*786 (13)*
**Highest level of care**			
A&E	580 (5)	262 (5)	306 (5)
Ward	6,323 (56)	1,697 (29)	3,701 (65)
Theatre	556 (5)	343 (5)	223 (4)
HC/MC	602 (5)	344 (6)	304 (5)
ICU	3,335 (29)	2,705 (47)	1,209 (21)
*Missing*	*899 (7)*	*412 (7)*	*482 (8)*

MMT = Mobile Medical Team, ICP = Intracranial pressure meter, A& = Accident and Emergency department, HC = High Care unit, MC = Medium Care unit, ICU = Intensive Care Unit.

Median ICU-LOS was 0 days (IQR: 0–2) , and H-LOS was 5 days (IQR: 2–11),—(Table 3). Thirteen per cent of patients died. Cyclists accounted for 49% of all deaths due to an RTA. Median number of days before they died was 3 (IQR: 2–7). Patients who survived had a median GOS of 4 (IQR: 4–5) at discharge, 88% had a GOS of 4 or 5 (n = 7,290), and 20% of GOS scores were missing. Most patients who survived were discharged to their usual place of residence (n = 6,438; 63%) (Table 3).

**Table 3:** Hospital outcome parameters.

	All	Polytrauma	Elderly
	n = 12,295	n = 5,763	n = 6,228
	**Median (IQR)**	**Median (IQR)**	**Median (IQR)**
GOS	4 (4–5)	4 (4–5)	4 (4–5)
*Missing*	*394 (4)*	94 (2)	*202 (4)*
H-LOS	5 (2–11)	7 (3–15)	5 (3–11)
*Missing*	*257 (2)*	*95 (2)*	*130 (2)*
I-LOS	0 (0–2)	1 (1–5)	0 (0–1)
*Missing*	*1,517 (12)*	*491 (9)*	*893 (14)*
LOS until death	3 (2–7)	3 (2–7)	3 (2–7)
*Missing*	*13 (1)*	*(1)*	*6 (1)*
	**N (%)**	**N (%)**	**N (%)**
Mortality	1,642 (13)	1,642 (28)	1,122 (18)
Discharge destination			
Usual place of residence	6,438 (63)	1,974 (46)	2,723 (56)
Rehabilitation centre	937 (9)	647 (15)	397 (8)
Nursing home	933 (9)	454 (11)	810 (17)
Care/residential home	197 (2)	89 (2)	167 (3)
Other	1,711 (17)	1,052 (25)	773 (16)
*Missing*	*437 (4)*	*208 (4)*	*236 (5)*

H-LOS = Length of stay in hospital, I-LOS = Length of stay in Intensive Care Unit, GOS = Glasgow Outcome Scale score.

### Polytrauma

There were 5,763 polytrauma patients, this was 47% of all moderate and severe TBI patients in our cohort. The incidence of polytrauma patients with moderate or severe TBI was 14 per 100,000 person-years. They had a median age of 64 years (IQR: 45–78), were predominantly male (n = 2092; 64%), with a median AIShead of 4 (IQR: 3–5) (Table 1).

Falls were the most common trauma mechanism (n = 2,533; 44%), closely followed by road traffic accidents (n = 2,249; 39%). Road traffic accidents (RTA) included cyclists, who accounted for a large proportion of this group (1,152 people, 51% of all RTAs), followed by motorised vehicles with more than two wheels (n = 452, 20%), mopeds (n = 322, 14%) and pedestrians (n = 205, 9%). Motorcycle accidents were less common (n = 85, 4%). Median RTS in A&E was 7.8 (5.0–7.8) (Table 1).

Both the MMT and in-hospital trauma teams were involved more often for these polytrauma patients, respectively in 1,514 (26%) and 3,161 (62%) of cases. Highest level of care was most often ICU (n = 2,705; 47%) (Table 2).

Median ICU-LOS was one day (IQR: 0–5) and H-LOS 7 days (IQR: 3–15), in ICU Fatalities were more frequent (n = 1,386; 24%). Median number of days before death was three (IQR: 2–7). Patients who survived had a median GOS of 4 (IQR: 4–5). Most patients who survived were discharged to their usual place of residence (n = 1,974; 46%) (Table 3).

### Elderly

There were 6,228 elderly patients, which is 51% of our cohort. The incidence rate of elderly patients is 67 per 100,000 person-years. They had a median age of 79 years (IQR: 72–85), 52% (n = 3,264) were male with a median AIShead of 3 (IQR: 3–4). (Table 1).

Falls were the most common trauma mechanism (n = 3,543; 57%), followed by RTA (n = 1,574; 25%), consisting mostly of bicycle accidents (n = 1,155; 73%), followed by pedestrians (n = 156, 10%), mopeds (n = 149, 9%) and accidents involving motorised vehicles with more than two wheels (n = 109, 7%). Motorcycle accidents were extremely rare (n = 5, < 1%). Of all falls, most were from low height, 85%. Median RTS in A&E was 7.8 (IQR: 7.6–7.8) (Table 1).

MMT and in-hospital trauma team were involved in 568 (10%) and 1,549 (31%) of cases, respectively. Highest level of care was most often on the ward (n = 3,702; 59%) (Table 2).

Median ICU-LOS was 0 days (0–1), H-LOS was 5 (IQR: 3–11) days.. Mortality rate was 18%, (n = 1,122). Median number of days before patients died was 3 (IQR: 2–7). Patients who survived had a median GOS of 4 (IQR: 4–5). Most patients who survived were discharged to their usual place of residence (n = 2,723; 46%) (Table 3).

## Discussion

There were over 12,000 patients with moderate or severe traumatic brain injury in this nationwide cohort, an incidence rate of 30/100,000 person-years. Patients in our cohort had a median age of 65 years, were predominantly male and were most often discharged home. Thirteen per cent of patients died. Patients who survived most often had good outcomes, with a median GOS of 4, this was even true for the elderly and polytrauma patients. Falls occurred more often than RTAs. Bicycle accidents were a commonly found trauma mechanism, especially amongst the elderly population. Moderate and severe TBI is far more common for the elderly, than the overall study population. Mortality in elderly was 18%, which was higher than the overall mortality, but less than for polytrauma patients (24%).

Falls are currently the most common cause of TBI in the USA and Germany^14^. Ever since the introduction of preventative measures, such as the mandatory seatbelt in cars and helmets for motorcyclists, fewer TBIs have been seen due to RTAs^21^. The epidemiology is changing with a relative increase of TBI amongst older patients, especially due to falls^14,22,23^. Amongst older patients, we see relatively more women, even though the stereotypical TBI patient used to be the young male^14,23^. The increase in TBI, however, cannot be attributed to aging alone^8,23^. For example, one study showed relatively more (mental) comorbidities and pre-injury hospital admissions amongst TBI patients, when matched for age, sex and postcode suggesting these comorbidities can lead to sustainment of TBI^24^.

Perhaps surprisingly, the most common cause of TBI in our polytrauma patients was also falls. RTAs were less common in the elderly, with 25%, but still account for almost 40% of TBI in polytrauma patients. More than half of RTAs consisted of bicycle accidents. Statistics show that deaths due to bicycle accidents have hardly decreased since 1996, as opposed to deaths due to car accidents^25^. In addition, almost half of our elderly patients had an ISS over 16 and almost half of polytrauma patients were elderly. This means that elderly patients do not necessarily have less severe injuries. Interestingly, a similar study in Germany found that patients who suffered a motor vehicle or motorcycle accident were much younger compared to their cohort overall^15^. The elderly in our cohort did suffer more from bicycle accidents than the overall cohort (73% vs 59%), and more than half of deaths due to bicycle accidents in 2018 were over 70 years old, which could indicate a similar phenomenon in the Netherlands^25^. Looking at these mechanisms, there are multiple options for prevention of moderate and severe TBI. For example, the large amount of cycling injuries could (re)start the debate on mandatory helmet use. In addition, the e-bike is gaining popularity amongst the elderly in the Netherlands and injuries from e-bike accidents are more severe than for regular bicycles and less than 1% wears a helmet^26^. Helmets could prevent TBI or at least lower the chances of severe TBI and need for neurosurgical intervention for cyclists^27^. Furthermore, fall prevention in the elderly population, could also lead to a decrease in the incidence of moderate and severe TBI.

The incidence of moderate and severe TBI found in this study is high and equates to approximately 30% of the incidence of lung cancer in the Netherlands^28^. Incidence is high when compared to the aforementioned German study as well, which also used a national trauma database. However, they used a Revised Injury Severity Classification score (RISC) score to classify TBI, and only included patients who were admitted to ICU or high intensity or medium care. Only 34% (n = 3,937) of our cohort received that level of care. This equates to a lower incidence rate of moderate and severe TBI admissions needing ICU admission than in Germany. Possibly, patients in that cohort were more severely injured which could also explain the much higher mortality rate of 23.5% in Germany. However, caution should be exercised in comparing our data to the German data since the RISC score used in the German study was found to be of limited predictive value in patients with moderate to severe TBI^29^.

Some patients made a full recovery, but remaining dependent on others in daily life is not uncommon^7^. Persistent disorders of consciousness, such as unresponsive wakefulness syndrome (UWS), where the patient does not demonstrate any sign of consciousness, can also occur as a result of TBI^7,30^. UWS is a rare phenomenon in the Netherlands^30^. Only 62 (0.5%) patients left hospital in this status in this cohort. Combined with the relatively short length of stay in ICU, this might lead to the conclusion that end of life decisions are taken quite early in admission for TBI patients. However, this seems to lead to a reduction in patients with poor outcomes, as outcome amongst patients who survived TBI were good in our cohort. Median GOS was 4 overall and for both subgroups and almost 90% of patients who survived their injuries had a GOS of 4 or 5. This was lower in the German study, with 61% of patients with a GOS of 4 or 5^14^. This phenomenon was suggested by an earlier retrospective study in the UMC Utrecht as well^7^.

Overall, highest level of care was the ward for most patients, polytrauma patients being the exception. Patients cared for on the ward, however, consist of two groups: patients who did not require ICU admission or patients who are not deemed fit for ICU admission and therefore received ward-based care. Of elderly patients, 69% had the ward as their highest level of care, but their mortality rate was slightly higher than for all patients (18% vs 13%). A ward-based care policy, meaning no cardiopulmonary resuscitation, intubation or ICU admission, may have contributed to this, perhaps as a result of therapeutic nihilism^8^. Less frequent involvement of the MMT and trauma team in initial care for the elderly, might also indicate that severity of TBI in the elderly is not always recognised before admission, possibly due to a low-impact trauma mechanism such as a fall from low height. This is supported by a Dutch study that investigated diagnostic value of pre-hospital emergency medical service providers^31^. Recognition of moderate or severe TBI in mainly elderly patients before admission, could therefore be a target for improvement as well.

The large database, covering all hospitals in the Netherlands is a major strength of this study as it is definitely representative of the whole country. Furthermore, the collected data is relevant to the epidemiology of TBI and most important factors can be found in the data.

This study has some limitations as well. Firstly, we decided not to impute for missing data, as this was a study designed to describe our population and their characteristics and our numbers were large enough to achieve this. In addition, sometimes the fact that data is missing can bring forward a new conclusion, for example, GCSs were poorly collected in this database, with only 75% available. More than half of patients had a GCS of 15, which is most likely not representative of our study population. In addition, even though alcohol and drugs intoxication can be of great impact on the nature of TBI, our database did not account for this. The same is applicable for the use of anticoagulants, as many elderly patients use these and they have a negative effect on TBI^22^. Therefore, we could not identify their role in our study population or recommend regulations, or stricter indication, regarding their use. In addition, the DTR uses the Glasgow Outcome Scale rather than the Glasgow Outcome Scale Extended, even though the latter is more sensitive^32^. Lastly, bicycles are an important mode of transport in the Netherlands. Therefore their contribution to moderate and severe TBI patients may well be different in other countries, as bicycles are not used as much and their position in traffic is different.

The fact that we used the AIS rather than GCS to classify TBI as moderate or severe, could potentially be seen as a limitation. The large amount of patients with a GCS of 15 included in our database, may support the theory that AIShead overscores severity of TBI, rather than poor data collection^33^. However, even a study who only included patients with a GCS of 3, still identified patients with mild or moderate TBI^12^. Furthermore, other studies have shown that GCS does not correlate well with the presence of TBI in elderly patients, who form a big part of our cohort^34,35^. As stated in our introduction, many external factors can influence GCS, such as intoxication and sedation, which make GCS less reliable^12,13^. The choice for AIS to determine severity of brain injury, rather than GCS, can make it difficult to compare our results to other studies. It seems injury is classified differently by the two parameters. The AIS can overscore injuries, when compared to the GCS^33^. A different study showed that a GCS of 3–8 predicted death better than an AIS of 5 or above in case of multiple injuries, but worse with isolated TBI^36^. This could be explained by the fact that GCS can be influenced by injuries in other regions of the body as well^36^. The AIS, however, remains one of the most common modes of classifying TBI and the gold standard of classifying traumatic injuries in general and is used commonly in retrospective data research^33^. Lastly, as the DTR does not regulate who will calculate the GCS and when in the resuscitation process this has happened, the AIShead seemed a more objective parameter for this study. Unfortunately, even the AIS is not completely resistant to inter-interpreter variability, as coding can be a difficult process, with interrater variability, although recent research showed that reliability for AIS coding in the DTR was substantial^18^. It would be preferable to have a more accurate system to classify TBI than GCS or AIS and we feel more research in this area is needed to allow for standardised research.

In conclusion, a change in the epidemiology of TBI occurred in the Netherlands, even for moderate and severe TBI as defined by the AIS: a shift to more elderly patients. Most common cause of moderate or severe TBI was falls, followed closely by RTAs. Bicycle accidents were very frequent, even more so amongst the elderly, suggesting prevention could be an important aspect in order to decrease morbidity and mortality by TBI.

## Data Availability

The data that support the findings of this study are available from the Dutch Trauma Registry, but restrictions apply to the availability of these data, which were used under license for the current study, and so are not publicly available. Data are however available from the authors upon reasonable request and with permission of the Dutch Trauma Registry.

## References

[1] Ghajar J (2000). Traumatic brain injury. Lancet.

[2] Taylor CA, Bell JM, Breiding MJ, Xu L (2017). Traumatic brain injury-related emergency department visits, hospitalizations, and deaths—United States, 2007 and 2013. MMWR. Surveill. Summ..

[3] Majdan M (2016). Epidemiology of traumatic brain injuries in Europe: A cross-sectional analysis. Lancet Public Heal..

[4] Van Den Brand CL (2018). Traumatic brain injury in the Netherlands, trends in emergency department visits, hospitalization and mortality between 1998 and 2012. Eur. J. Emerg. Med..

[5] Gunning AC (2015). Demographic patterns and outcomes of patients in level I trauma centers in three international trauma systems. World J. Surg..

[6] Jochems D, Leenen LPH, Hietbrink F, Houwert RM, van Wessem KJP (2018). Increased reduction in exsanguination rates leaves brain injury as the only major cause of death in blunt trauma. Injury.

[7] Jochems D (2018). Outcome in patients with isolated moderate to severe traumatic brain injury. Crit. Care Res. Pract..

[8] Maas AIR (2017). Traumatic brain injury: integrated approaches to improve prevention, clinical care, and research. Lancet Neurol..

[9] Roozenbeek B, Maas AIR, Menon DK (2013). Changing patterns in the epidemiology of traumatic brain injury. Nat. Rev. Neurol..

[10] Johnson WD, Griswold DP (2017). Traumatic brain injury: A global challenge. Lancet Neurol.

[11] Scholten AC, Haagsma JA, Panneman MJM, Van Beeck EF, Polinder S (2014). Traumatic brain injury in the Netherlands: Incidence, costs and disability-adjusted life years. PLoS ONE.

[12] Salottolo K (2017). The epidemiology, prognosis, and trends of severe traumatic brain injury with presenting Glasgow Coma Scale of 3. J. Crit. Care.

[13] Jonsdottir GM (2017). A population-based study on epidemiology of intensive care unit treated traumatic brain injury in Iceland. Acta Anaesthesiol. Scand..

[14] Maegele M (2019). Inzidenz und Versorgung des mittelschweren bis schweren Schädel-Hirn-Traumas. Dtsch. Arztebl. Int..

[15] Peek J (2020). Epidemiology and outcome of rib fractures: A nationwide study in the Netherlands. Eur. J. Trauma Emerg. Surg..

[16] Kuipers, E. J. & Leenen, L. P. H. Landelijke traumaregistratie 2013–2017. (2018). Available from: https://www.lnaz.nl/cms/files/lnaz_ltr_jaarrapport_2014-2018__november_2019.pdf

[17] Centraal bureau voor statistiek (CBS). Bevolking; geslacht; leeftijd en burgerlijke staat, 1 januari. 2020. Available from: https://opendata.cbs.nl/statline/#/CBS/nl/dataset/7461BEV/table?fromstatweb.

[18] Olthof DC, Luitse JSK, De Groot FMJ, Goslings JC (2013). A Dutch regional trauma registry: Quality check of the registered data. BMJ Qual. Saf..

[19] Gennarelli, T. A., & Wodzin, E. *Abbreviated Injury Scale 2005. Update 2008. Association for the Advancement of Automotor Medicine* (Barrington, IL, 2008).

[20] Van Camp LA, Deloooz HH (1998). Current trauma scoring systems and their applications. Eur. J. Emerg. Med..

[21] Di Saverio S (2014). Changes in the outcomes of severe trauma patients from 15-year experience in a Western European trauma ICU of Emilia Romagna region (1996–2010). A population cross-sectional survey study. Langenbeck’s Arch. Surg..

[22] Schumacher, R., Müri, R. M. & Walder, B. Integrated health care management of moderate to severe TBI in older patients—A narrative review (2017). 10.1007/s11910-017-0801-7 28986740

[23] Peeters W, Majdan M, Brazinova A, Nieboer D, Maas AIR (2017). Changing epidemiological patterns in traumatic brain injury: A longitudinal hospital-based study in Belgium. Neuroepidemiology.

[24] Lystad RP, Cameron CM, Mitchell RJ (2019). Excess mortality among adults hospitalized with traumatic brain injury in Australia: A population-based matched cohort study. J. Head Trauma Rehabil..

[25] Centraal bureau voor statistiek (CBS). Overledenen; doden door verkeersongeval in Nederland, wijze van deelname. 1–2 (2016). Available from: http://statline.cbs.nl/StatWeb/publication/?VW=T&DM=SLNL&PA=71936ned&LA=NL

[26] Poos HPAM (2017). E-bikers are more often seriously injured in bicycle accidents: Results from the Groningen bicycle accident database. Ned. Tijdschr. Geneeskd..

[27] Dodds N (2019). Evaluating the impact of cycle helmet use on severe traumatic brain injury and death in a national cohort of over 11000 pedal cyclists: A retrospective study from the NHS England Trauma Audit and Research Network dataset. BMJ Open.

[28] Nederlandse Kanker Registratie. Incidentie Longkanker. [Internet]. Available from: ttp://iknl/nkr-cijfers. [Accessed on 14th June 2020].

[29] Raj R (2015). Validation of the revised injury severity classification score in patients with moderate-to-severe traumatic brain injury. Injury.

[30] Van Erp WS (2015). The vegetative state: prevalence, misdiagnosis, and treatment limitations. JMDA.

[31] van Rein EAJ (2018). Diagnostic value of emergency medical services provider judgment in the identification of head injuries among trauma patients. Eur. J. Neurol..

[32] McMillan T (2016). The Glasgow outcome scale-40 years of application and refinement. Nat. Rev. Neurol..

[33] Rogers S, Trickey AW (2017). Classification of traumatic brain injury severity using retrospective data. J. Nurs. Educ. Pract..

[34] Rozenfeld M (2020). The reliability of the Glasgow Coma Scale in detecting traumatic brain injury: The continuous effect of age. Brain Inj..

[35] Salottolo K, Stewart Levy A, Slone DS, Mains CW, Bar-Or D (2014). The effect of age on glasgow coma scale score in patients with traumatic brain injury. JAMA Surg..

[36] Savitsky B, Givon A, Rozenfeld M, Radomislensky I, Peleg K (2016). Traumatic brain injury: It is all about definition. Brain Inj..

